# Molecular Docking, Bioinformatic Analysis, and Experimental Verification for the Effect of Naringin on ADHD: Possible Inhibition of GSK-3β and HSP90

**DOI:** 10.3390/ph17111436

**Published:** 2024-10-26

**Authors:** Hatem I. Mokhtar, Sawsan A. Zaitone, Karima El-Sayed, Rehab M. Lashine, Nada Ahmed, Suzan M. M. Moursi, Shaimaa A. Shehata, Afaf A. Aldahish, Mohamed A. Helal, Mohamed K. El-Kherbetawy, Manal S. Fawzy, Noha M. Abd El-Fadeal

**Affiliations:** 1 Department of Pharmaceutical Chemistry, Faculty of Pharmacy, Sinai University-Kantara Branch, Ismailia 41636, Egypt; hatem.mokhtar@su.edu.eg; 2 Department of Pharmacology & Toxicology, Faculty of Pharmacy, University of Tabuk, Tabuk 47713, Saudi Arabia; 3 Department of Pharmacology & Toxicology, Faculty of Pharmacy, Suez Canal University, Ismailia 41522, Egypt; 4 Medical Physiology Department, Faculty of Medicine, Suez Canal University, Ismailia 41522, Egypt; 5 Clinical Pharmacology Department, Faculty of Medicine, Suez Canal University, Ismailia 41522, Egypt; 6 Department of Clinical Pathology, Faculty of Medicine, Suez Canal University, Ismailia 41522, Egypt; 7 Medical Physiology Department, Faculty of Medicine, Zagazig University, Zagazig 44519, Egypt; 8 Department of Forensic Medicine and Clinical Toxicology, Faculty of Medicine, Suez Canal University, Ismailia 41522, Egypt; 9 Department of Pharmacology, College of Pharmacy, King Khalid University, Abha 61441, Saudi Arabia; adahesh@kku.edu.sa; 10 Biomedical Sciences Program, University of Science and Technology, Zewail City of Science and Technology, October Gardens, 6th of October, Giza 12587, Egypt; mohamed.hilal@pharm.suez.edu.eg; 11 Medicinal Chemistry Department, Faculty of Pharmacy, Suez Canal University, Ismailia 41522, Egypt; 12 Department of Pathology, Faculty of Medicine, Suez Canal University, Ismailia 41522, Egypt; mohamed_elkherbetawy@med.suez.edu.eg; 13 Department of Biochemistry, Faculty of Medicine, Northern Border University, Arar 91431, Saudi Arabia; manal.darwish@nbu.edu.sa; 14 Medical Biochemistry and Molecular Biology Department, Faculty of Medicine, Suez Canal University, Ismailia 41522, Egypt; 15 Biochemistry Department, Ibn Sina National College for Medical Studies, Jeddah 22421, Saudi Arabia

**Keywords:** ADHD, mouse, monosodium glutamate, naringin, Wnt/β-catenin signaling, molecular docking

## Abstract

**Background/Objectives:** One of the most abundant and growing neurodevelopmental disorders in recent decades is attention deficit hyperactivity disorder (ADHD). Many trials have been performed on using drugs for the improvement of ADHD signs. This study aimed to detect the possible interaction of naringin with Wnt/β-catenin signaling and its putative anti-inflammatory and protective effects in the mouse ADHD model based on bioinformatic, behavioral, and molecular investigations. Furthermore, molecular docking was applied to investigate possible interactions with the GSK-3β and HSP90 proteins. **Methods**: Male Swiss albino mice were divided into four groups, a normal control group, monosodium glutamate (SGL) control, SGL + naringin 50 mg/kg, and SGL + naringin 100 mg/kg. The psychomotor activity of the mice was assessed using the self-grooming test, rope crawling test, and attentional set-shifting task (ASST). In addition, biochemical analyses were performed using brain samples. **Results**: The results of the SGL group showed prolonged grooming time (2.47-folds), a lower percentage of mice with successful crawling on the rope (only 16.6%), and a higher number of trials for compound discrimination testing in the ASST (12.83 ± 2.04 trials versus 5.5 ± 1.88 trials in the normal group). Treatment with naringin (50 or 100 mg per kg) produced significant shortening in the grooming time (31% and 27% reductions), as well as a higher percentage of mice succeeding in crawling with the rope (50% and 83%, respectively). Moreover, the ELISA assays indicated decreased dopamine levels (0.36-fold) and increased TNF-α (2.85-fold) in the SGL control group compared to the normal mice, but an improvement in dopamine level was observed in the naringin (50 or 100 mg per kg)-treated groups (1.58-fold and 1.97-fold). Similarly, the PCR test showed significant declines in the expression of the Wnt (0.36), and β-catenin (0.33) genes, but increased caspase-3 (3.54-fold) and BAX (5.36-fold) genes in the SGL group; all these parameters were improved in the naringin 50 or 100 mg/kg groups. Furthermore, molecular docking indicated possible inhibition for HSP90 and GSK-3β. **Conclusions**: Overall, we can conclude that naringin is a promising agent for alleviating ADHD symptoms, and further investigations are required to elucidate its mechanism of action.

## 1. Introduction

Attention deficit hyperactivity disorder (ADHD) is a juvenile neurological illness affecting approximately 2.5% of children, with many individuals continuing to exhibit symptoms into adulthood [1,2]. The condition is characterized by behaviors that disrupt social interactions, including hyperactivity, impulsiveness, and inattention, which can significantly impact educational outcomes and personal development [3]. Environmental factors such as hypoxia during prenatal life may also play a role in the development of ADHD [4]. Evidence from imaging, clinical, and experimental studies documented the involvement of catecholamine dysregulations in ADHD. For example, amplified dopamine transporter binding was observed in ADHD patients [5,6], and reduced noradrenaline transporter expression in specific brain regions [7].

Recent studies have highlighted the significance of protein targets such as heat shock protein 90 (HSP90) and glycogen synthase kinase 3 beta (GSK-3β) in the context of neurodevelopmental disorders, including ADHD [8,9]. HSP90 is involved in the regulation of various signaling pathways and is crucial for the stabilization and proper functioning of several proteins involved in neurotransmission [10]. Dysregulation of HSP90 has been linked to neurodevelopmental disorders, suggesting its potential role in the pathophysiology of ADHD [8]. Recent studies showed that HSP90 inhibition prevented neuronal cell loss [11,12] and rescue synaptic dysfunction in animal models of neurodegeneration [13]. Further, HSP inhibition protects against retinal degeneration in rats [14] and open-angle glaucoma [15].

Additionally, GSK-3β is known to be involved in neurodevelopment and synaptic plasticity; its dysregulation can impact dopamine signaling and is implicated in the clinical manifestations of ADHD [16,17]. These findings provide a compelling rationale for exploring interventions that target these proteins in the context of ADHD. Exposure to glycogen synthase kinase 3 (GSK3) inhibitors significantly increased activation of Wnt/β-catenin signaling [18]. Chemical screening efforts have prioritized GSK-3β inhibitors as inducers of cell differentiation [19]. Ruiz and Eldar-Finkelman reviewed the use of GSK-3 inhibitors in CNS disorders [20]. Hence, legends that possess an inhibiting activity against Hsp90 are promising neuroprotectants.

The Wnt/β-catenin is an evolutionarily developmental signaling pathway that plays a crucial role in tissue homeostasis [21]. The Wnt/β-catenin signaling pathway has been associated with the etiology of various diseases, including ADHD [22,23,24]. Aberrant Wnt signaling may affect neuronal development and function, further contributing to ADHD symptomatology [25].

Psychostimulants such as amphetamine are first-line pharmacotherapies for patients with ADHD [26]. The longer-acting stimulants deliver extended effectiveness, which restricts the need for daily dosing and lessens the social stigma related to taking medications in the school setting. However, they result in side effects and are more costly [27]. The shorter-duration drugs require high compliance, since they should be taken 2–3 times a day [28]. On the other hand, non-stimulant drugs have the advantage of reduced liability for substance misuse. For example, atomoxetine has a lower potential for abuse, but its efficacy is lower than stimulants. Atomoxetine carries a warning for increasing suicidal potential or the development of jaundice or liver injury [28,29]. There are also alpha agonists, such as clonidine and guanfacine, which activate the CNS presynaptic autoreceptor inhibitory function. These drugs have some disadvantages, such as requiring multiple daily doses, and may result in a hypotensive effect [29]. Another drug is bupropion, which works through dopamine and norepinephrine, and we can expect side effects upon its use, such as irritability, anorexia, insomnia, and increased risk for seizures [28]

Monosodium glutamate (SGL) is a common food flavor enhancer agent used on a wide scale [30]. Improper consumption of SGL above the allowed level is documented to raise glutamate blood concentration, cross the blood–brain barrier, and accumulate in CNS tissue [31]. Exaggerated glutamate levels are known as causes of excitotoxicity and are reported to induce many neurodegenerative disorders, including ADHD [32,33]. SGL induces persistent neurotoxic impact by increasing oxidative stress injury, apoptosis, and neurodegeneration [34,35].

Naringin (4′,5,7-trihydroxy flavanone 7-rhamnoglucoside) is an abundant flavanone in grapefruit and Citrus species [36,37]. Upon oral administration, naringin hydrolyzes to naringenin. The latter is a major metabolite that can be absorbed easily from the GIT [37]. Naringin can cross the blood–brain barrier [38] and was documented to possess anti-inflammatory, anti-oxidative, and anti-apoptotic properties in rat hippocampi [39]. Naringin has been considered a neuroprotective agent due to the induction of neurotrophic factors [40]. Furthermore, naringin exhibited a neuroprotective effect against neuronal apoptosis through modulation of the Bax and Bcl-2 pathways [41]. Collectively, naringin acts on several neuroprotective antioxidants that function against oxidative neurotoxicity in vitro [42] and hippocampal oxidative neurotoxicity in rats [43]. Further, Ahmed et al. reviewed the therapeutic potential of naringin in neurologic disorders and suggested further study and consideration of this compound as a potential candidate for neurotherapeutics [44].

Currently, there is still an inadequate understanding of the influence of nutraceuticals in alleviating ADHD symptoms. Hence, the current study was designed to investigate the ameliorative effect of naringin on the attentive behavior of an ADHD mouse model induced by SGL. The aim was extended to exploring the possible inhibition of HSP90 and GSK-3β by naringin, leading to increasing Wnt/β-catenin signaling.

## 2. Results

### 2.1. Molecular Docking and Bioinformatic Results

**Figure 1:**
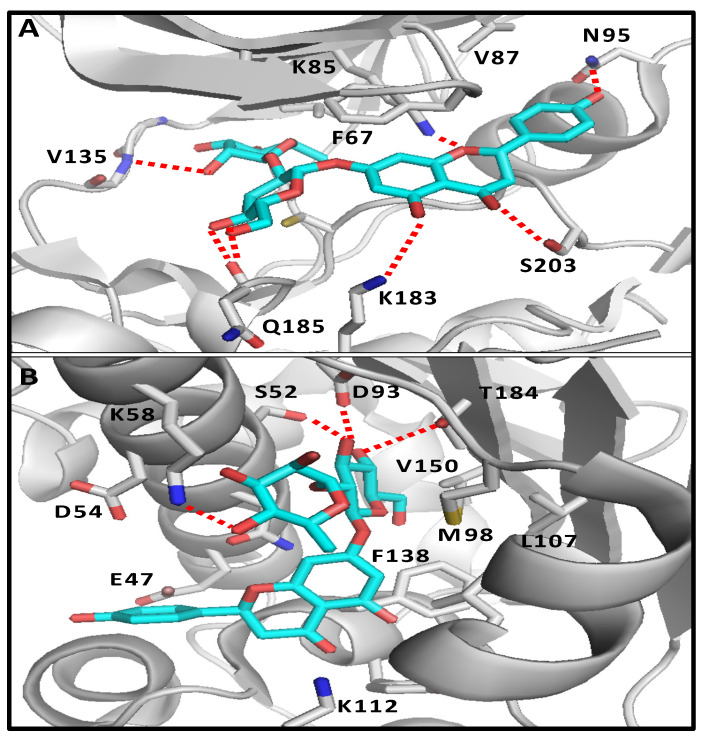
Proposed binding mode of naringin in the ATP-binding site of GSK-3β (**A**) and HSP90 (**B**). Ligand is displayed as cyan sticks and the important protein residues are displayed as gray sticks with a cartoon backbone. Polar contacts are shown as red dashed lines.

Naringin was docked into the active site of its potential targets, GSK-3β and HSP90. The proposed binding mode of naringin in the active site of GSK-3β (PDB ID: 4AFJ) shows the expected and essential H-bond between the disaccharide moiety and the backbone of Val135 in the hinge region of the kinase [45]. In addition, the aglycone part fits nicely into the large hydrophobic back pocket of the enzyme, making several contacts. As depicted in Figure 1A, these interactions include two strong H-bonds with the catalytic Lys85 and Asn95, and relatively weak contacts with Lys183 and Ser203. It was noticed that the 2-phenyl-chromen-4-one scaffold places the four oxygens in an ideal orientation for these H-bonding interactions. In addition, the phenyl ring of the chromene nucleus forms a close π-stacking interaction with Phe67 at the top of the active site.

Additionally, we docked naringin into the ATP-binding site located centrally in the Hps90-NTD using the crystal structure of the protein in a complex with the potent triazole-based inhibitor, JMC31 (PDB ID: 8AGI) [46]. The disaccharide fragment shows H-bonds with the critical Asp93 and Thr184 in the ATP-binding site (Figure 1B) [46,47]. In addition, the chromene ring fits into the lower hydrophobic pocket, making a π-stacking interaction with Phe138 and favorable hydrophobic contacts with Leu107. Moreover, it is worth noting that the naringin carbonyl lies in close proximity to the critical Lys112, which is essential for binding with ATP, and could be involved in a water-mediated H-bond with this important residue [47]. Further, the phenolic ring of naringin extends into the solvent-exposed pocket in a similar fashion to the cyclohexyl ring of JMC31.

**Figure 2:**
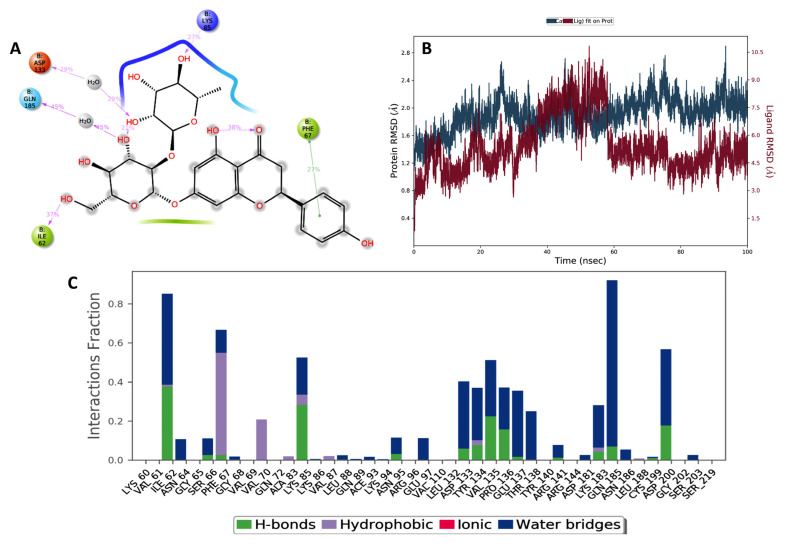
(**A**) Two-dimensional representation of the binding mode of naringin in the ATP-binding site of GSK-3β. The interaction stability over the MD simulation is displayed as a percentage beside each interaction. (**B**) RMSD of the backbone and the ligand during the MD simulation. (**C**) Interaction fraction diagram during the 100 ns simulation.

In addition, a molecular dynamics (MD) simulation was performed to study the time-dependent behavior of the complexes and validate the proposed binding modes (Figure 2A). For the naringin-GSK-3β complex, and as evident from the RMSD chart (Figure 2B), the protein backbone slightly fluctuated in the beginning of the simulation and then stabilized after 40 ns, while the ligand showed a significant fluctuation around 40–60 ns and remained stable close to its initial orientation until the end of the simulation run. Figure 2B represents the predominant interactions and their stability during the simulation time. It is evident that the critical H-bond with the hinge region residue Val135 was stable for more than 50% of the MD time, either directly or via a water molecule. Interestingly, the interaction fraction diagram showed H-bonding with Lys85 and Lys183, where the most significant polar contacts contribute to the binding in the hydrophobic pocket and might be responsible for the binding pose stability.

**Figure 3:**
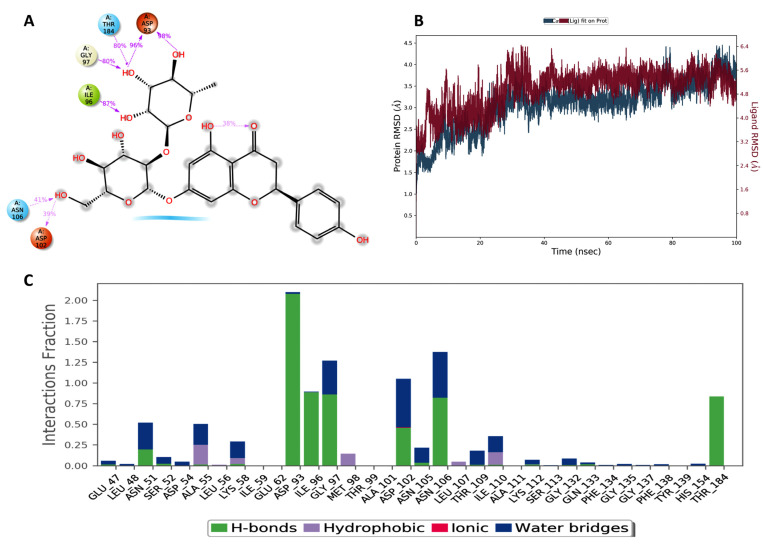
(**A**) Two-dimensional representation of the binding mode of naringin in the ATP-binding site of HSP90. The interaction stability over the MD simulation is displayed as a percentage beside each interaction. (**B**) RMSD of the backbone and the ligand during the MD simulation. (**C**) Interaction fraction diagram during the 100 ns simulation.

On the other hand, the complex of naringin with HSP90 showed the expected stable H-bonds with Asp93 and Thr184, as described above, with persistent interactions for 96% and 80% of the simulation time, respectively (Figure 3A). The protein backbone and the ligand showed fluctuation at the beginning of the simulation and then stabilized after 40 ns around 3.5 and 5.2 Å, respectively (Figure 3B). To our delight, the overall proposed binding mode was found to become tighter, with the ligand moving deeper into the ATP pocket, facilitated by the strong H-bond anchoring.

### 2.2. The Bioinformatic Results

**Figure 4:**
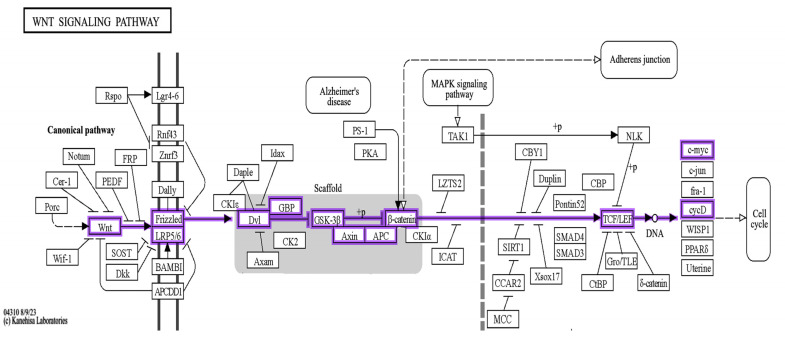
Wnt signaling pathway [04310]. The pathway was obtained from the KEGG database and shows that Wnt/β-catenin is involved in the nuclear translocation of β-catenin and the activation of target genes via TCF/LEF transcription factors, making this pathway crucial for the self-renewal of cells.

A search was performed on the KEGG pathway for the mechanism of action of naringin and found that the Wnt pathway is involved (map04310). It is known that the Wnt pathway is crucial for some of the morphogen production necessary for basic development. Integration between the naringin mechanism and the canonical Wnt pathway was noticed. Wnt protein inhibits the β-catenin degradation complex and, at this time, β-catenin becomes ready to enter the nucleus and stimulate the Wnt-controlled genes (Figure 4).

**Figure 5:**
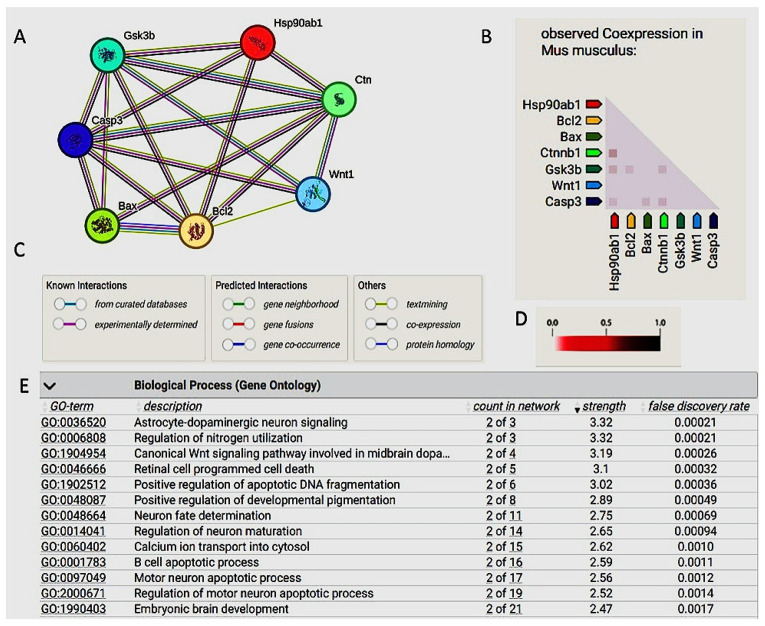
Role of Wnt//β-catenin signaling. (**A**) Wnt/β-catenin interactions with many proteins, such as GSK3B, HSP90, Casp3, BAX, and Bcl2. (**B**) Co-expression analysis of interacting proteins. (**C**) The colored lines indicate different evidence types. For example, red is fusion evidence, light blue is a database, green is neighborhood, blue is co-occurrence, purple is experimental evidence, black is co-expression, and yellow is text mining evidence. (**D**) The intensity of the color indicates the level of confidence that two proteins are functionally associated. (**E**) Gene ontology shows the molecular processes most associated with Wnt/β-catenin signaling. Wnt1: proto-oncogen Wnt-1, CASP3: caspase-3 subunit p-12, BAX: apoptosis regulator BAX, BCL2: BCL2-like protein 2, Hsp90ab1: heat shock protein HSP 90-beta1, Ctnnb1: catenin beta-1, Gsk3b: glycogen synthase kinase-3 beta.

A STRING database analysis of protein-protein interactions (PPI) reveals that Wnt/β-catenin proteins impact proteins involved in cell cycle regulation and apoptosis through interactions with several proteins, such as GSK-3β and HSP90ab1. These interactions adversely affect various cellular processes, including cell survival. Therefore, controlling the expression levels of these proteins may help to protect cells. The database revealed that the PPI enrichment is highly significant, with a *p*-value of 0.000591 (Figure 5A–D), and the gene ontology molecular process revealed its role in the regulation of neuronal cell maturation and brain development, with an evidence score = 2.47 (Figure 5E).

**Figure 6:**
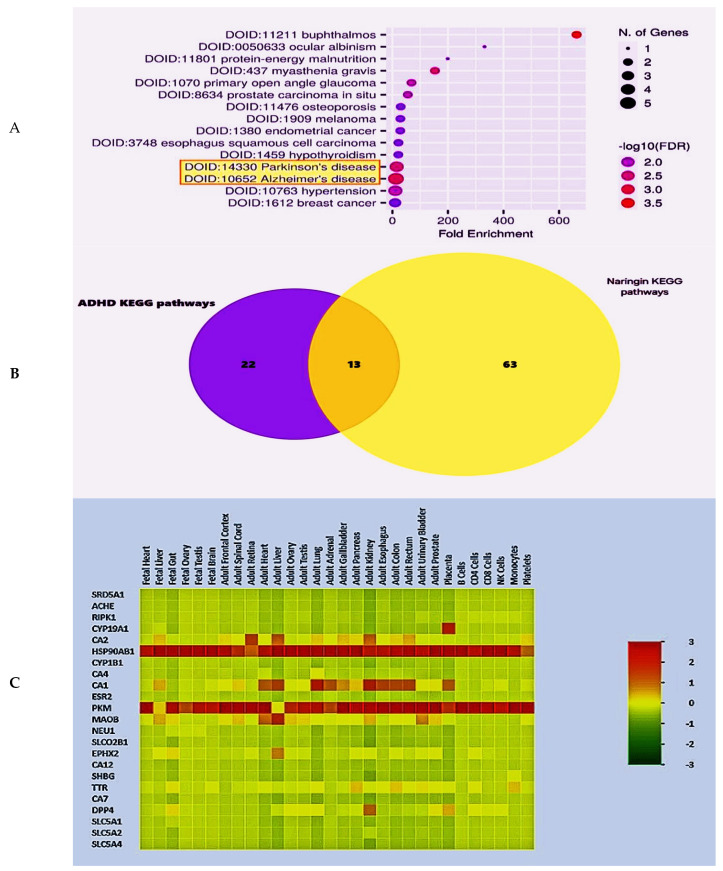
(**A**) A ShinyGo 0.8 dot plot representing the top 15 diseases related to naringin target genes. (**B**) A Venn diagram showing the target genes of naringin (yellow circle) and ADHD (violet circle) and the common pathways between them (brown part). The diagram was created using FunRich. (**C**) A heatmap demonstrating the model of gene expression of naringin target genes. The FunRich 3.1.3 bioinformatic tool was used. The color code of the heatmap ranges from 3 to −3, with 3 (most red) being the most expressed and −3 (most green) being the least expressed.

Furthermore, a dot plot was created for presenting the top fifteen diseases related to the designated target genes determined for naringin (Figure 6A). In addition,, the Disease. Alliance database was used with an FDR cutoff value of 0.05. Cognitive disorders, such as PD and AD, were found to have a strong relation with many of the naringin target genes (Figure 6A). To demonstrate these data, a Venn diagram was plotted using the FunRich 3.1.3 tool (Figure 6B); it shows the shared pathways between naringin and ADHD-related genes. Indeed, thirteen pathways were noticed in common between the naringin-related genes (originally 76) and the ADHD-related pathways (originally 35 pathways). Moreover, a heatmap was made with the aid of the FunRich 3.1.3. tool to present the pattern by which gene expression takes place (Figure 6C).

### 2.3. Mouse Study Results

#### 2.3.1. Self-Grooming and Rope Crawling Tests

**Figure 7:**
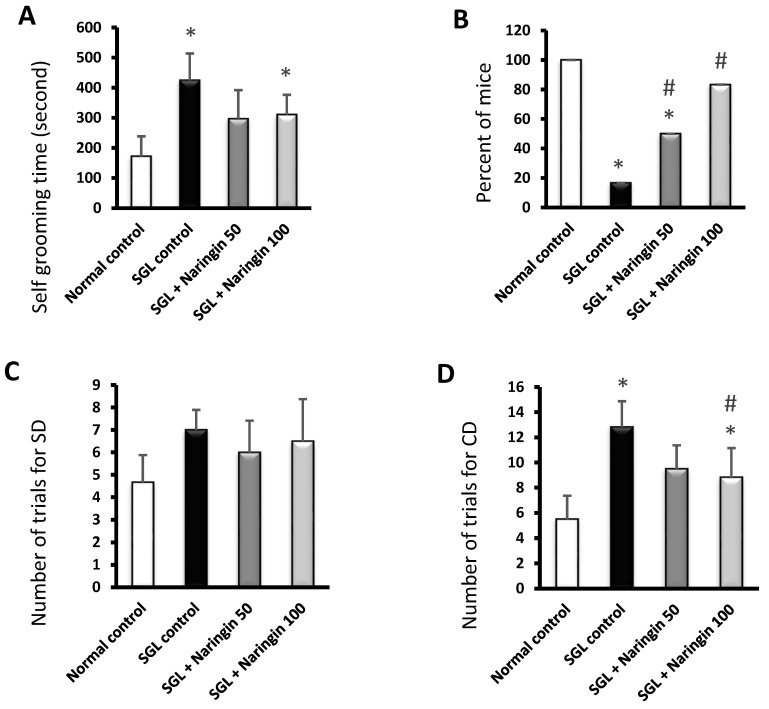

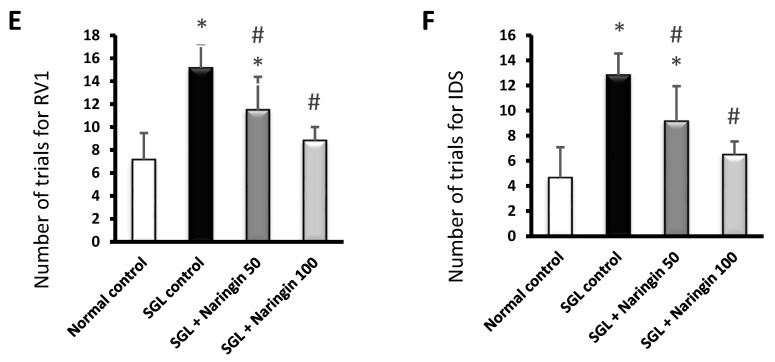
The behavior of mice in the self-grooming test and rope crawling test, and the number of trials in ASTT. (**A**) The time taken for self-grooming. (**B**) The percentage of mice (out of six) crawling on the rope. (**C**) The SD phase, (**D**) the CD phase, (**E**) the RV1 phase, and (**F**) the IDS phase. Data are means ± SD except for Panel (**B**), which is the % of animals. At *p* < 0.05, * vs. normal and # vs. SGL control.

The results of the self-grooming test indicated a prolongation of the grooming time in the SGL control group. On the other hand, the naringin-50- and naringin-100-treated groups did not show significant shortening in the grooming time, as indicated in Figure 7A. In addition, a low percentage of mice (16.66%) succeeded in crawling with the rope in the SGL control group. However, the naringin-50 and naringin-100 groups demonstrated high percentages of successful mice (50% and 83.33%), as shown in Figure 7B.

#### 2.3.2. ASST

In the ASST, the observed numbers of trials for SD were not different among the experimental groups (Figure 7C). The number of trials for CD in the SGL control was larger than in the normal control. The mice in the naringin-100 groups required fewer trials to achieve the CD criteria, as shown in Figure 7D. Further, the SGL control group consumed more RV1 and IDS criteria trials than the normal control group. The naringin-50 and naringin-100 groups showed significant declines in the required trials to achieve RV1 and IDS criteria (Figure 7E,F).

#### 2.3.3. ELISA Assays

**Figure 8:**
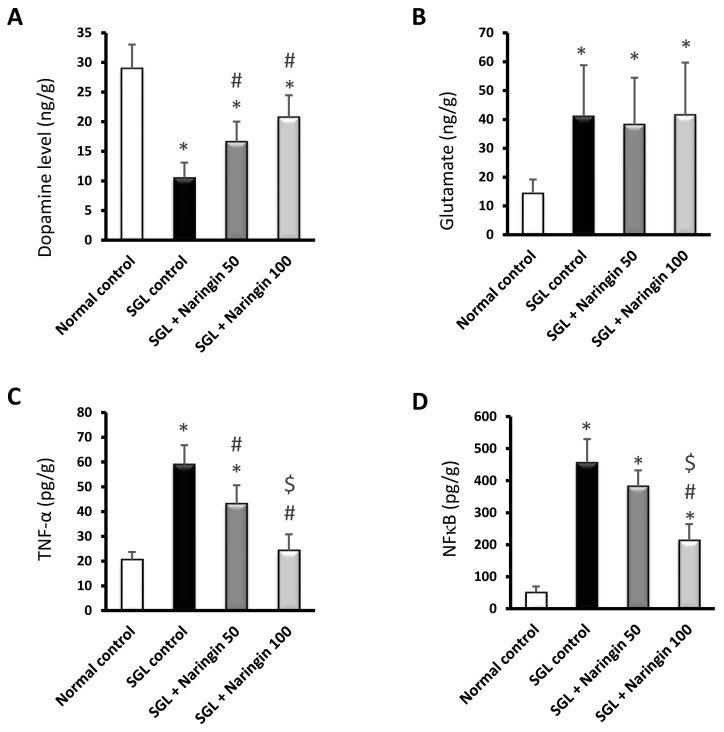
Naringin modulates the brain levels of glutamate, dopamine, and inflammation markers in mice fed with SGL. (**A**) Dopamine, (**B**) glutamate, (**C**) TNF-α, and (**D**) NFκB. Data are mean ± SD. At *p* < 0.05, * vs. normal, # vs. SGL control, and $ vs. SGL + naringin-50.

The current results indicate decreased dopamine content in the brains of the SGL control group compared with the normal control group (Figure 8A). In contrast, increased glutamate levels were observed in the SGL group (Figure 8B). The naringin-treated groups (50 and 100 mg) showed significant improvements in brain dopamine levels. However, these two groups did not show changes in glutamate levels compared to the SGL group. The TNF-α and NFκB levels in the brains were significantly elevated in the SGL control group versus the normal group. A dose-dependent decline in TNF-α levels was noticed in the naringin-treated groups (50 and 100 mg) (Figure 8C). However, NFκB was significantly reduced in the naringin (100 mg) group (Figure 8D).

#### 2.3.4. RT-PCR Analysis

**Figure 9:**
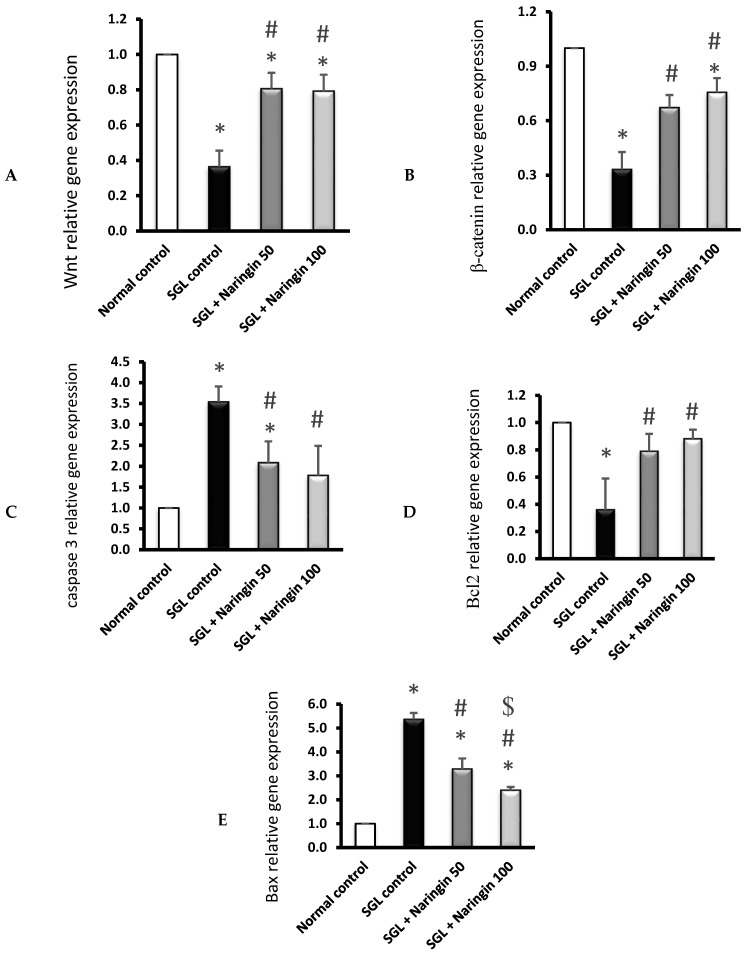
RT-PCR analysis of the expression of the target genes. (**A**) Wnt, (**B**) β-catenin, (**C**) caspase-3, (**D**) Bcl2, and (**E**) BAX. Data are means ± SD, * vs. normal control and # vs. SGL control, ^$^ vs. the SGL/naringin-50 group at *p* < 0.05.

The results of the current study indicated a significant reduction in the expression of the Wnt gene and the β-catenin gene in the SGL mouse group. Conversely, the treatment with naringin at 50 mg and 100 mg resulted in upregulated genes. Additionally, the levels of the apoptotic genes, caspase-3 and BAX, were upregulated in the SGL mouse group and downregulated in both naringin-treated groups (50 and 100 mg). However, a significant difference was found in the expression level of the BAX gene between the naringin 50 mg and naringin 100 mg/kg groups. Moreover, the anti-apoptotic gene Bcl2 was downregulated in the SGL group compared with the normal group. Interestingly, treatment with naringin at both 50 mg/kg and 100 mg/kg caused the upregulation of Bcl2 expression levels, and this may suggest a protective effect of naringin against apoptotic events (Figure 9A–E).

#### 2.3.5. Hematoxylin and Eosin Staining

**Figure 10:**
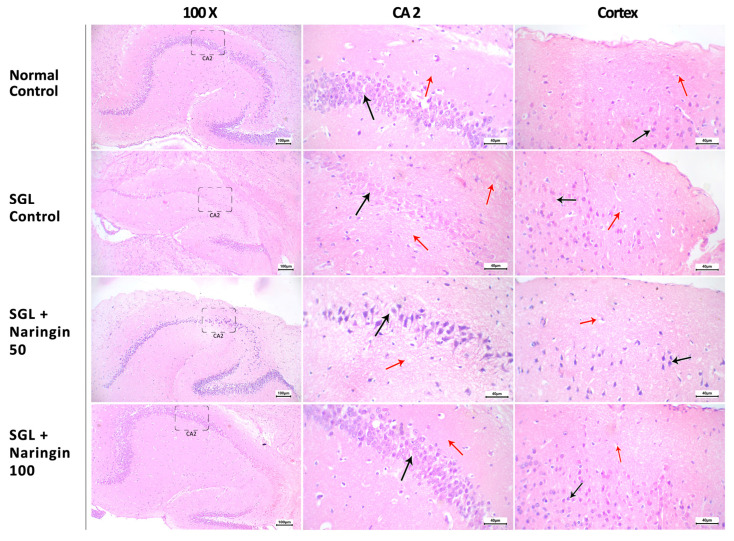
H&E-stained sections of the mice groups. The normal group shows neuron cell bodies arranged in a compacted form and regular nuclei, indicated by a black arrow, with intact fibrillary cytoplasmic processes (red arrow). The cortex shows normal neurons (black arrow) and astrocytic cells (red arrow). The SGL control CA2 region shows smudged nuclei of neurons with pericellular vacuolation and a mildly disrupted arrangement (black arrow) with decreased cellularity, and there are moderately disturbed fibrillary processes in multiple areas (red arrows). The cortex displays degenerate neurons (black arrow) and increased vacuolation of astrocytic cells (red arrow). The SGL + naringin 50 CA2 region shows focal pericellular vacuolation (black arrow) and scattered fibrillary process degeneration (red arrow). The cortex shows mild degenerative changes to neurons (black arrow) and mild vacuolation of astrocytic cells (red arrow). The SGL + naringin 100 CA2 region shows a regular arrangement of neurons with cell bodies showing normal chromatin patterns and nuclei with minimal vacuolation (black arrow), and intact fibrillary processes (red arrow). The cortex shows regular neurons (black arrow) with astrocytic cells showing minimal vacuolation (red arrow).

In Figure 10, H&E-staining is demonstrated at sections in the hippocampus. The normal group shows neuron cell bodies arranged in a compacted form, and regular nuclei with intact fibrillary cytoplasmic processes are shown. The cortex shows normal neurons and astrocytic cells. The SGL control CA2 region shows smudged nuclei of neurons with pericellular vacuolation and mildly disrupted arrangement with decreased cellularity, and there are moderately disturbed fibrillary processes in multiple areas. The cortex shows degenerate neurons and increased vacuolation of astrocytic cells. In the SGL + naringin 50, the CA2 region shows focal pericellular vacuolation and scattered fibrillary process degeneration. The cortex shows mild degenerative changes to neurons and mild vacuolation of astrocytic cells (red arrow). The SGL + naringin 100 CA2 region shows a regular arrangement of neurons with cell bodies showing normal chromatin patterns and nuclei with minimal vacuolation (black arrow), and intact fibrillary processes. The cortex shows regular neurons with astrocytic cells showing minimal vacuolation.

#### 2.3.6. Immunohistochemical Staining for Bcl2

**Figure 11:**
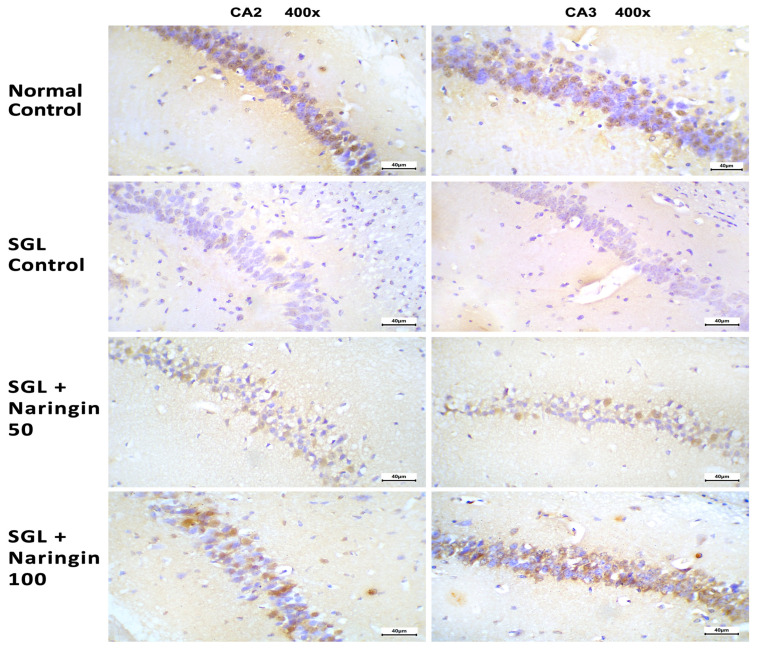
Immunohistochemical staining for Bcl2 in the hippocampi of the experimental groups. The normal group showed organized neurons with moderate-to-strong nuclear staining in most of the cells (CA2 and CA3 regions). The SGL control group showed less organized neurons with weak-to-absent staining for Bcl2 in most of the cells (CA2 and CA3 regions). The SGL + naringin-50 group showed mild-to-moderate staining in a few cells (CA2 and CA3 regions). The SGL + naringin-100 group showed improvements in the neuronal structures and moderate-to-strong staining for Bcl2 in most of the cells (CA2 and CA3 regions). Bcl2 immunostaining at ×400 magnification for both images in each group.

Figure 11 shows immunohistochemical staining in the hippocampi of the experimental groups. The normal control group showed organized neurons with strong cytoplasmic staining (first row). (However, the SGL control group showed less organized neurons with weak staining for Bcl2 (second row)). The SGL + naringin-50 group showed mild staining (third row), whereas the SGL + naringin-100 group showed moderate staining for Bcl2 (forth row).

## 3. Discussion

ADHD is a neurodevelopmental disorder with onset in childhood. Failure to undergo treatment causes symptoms to worsen and complications to develop, including low self-esteem, school failures, depression, and addictions. In ADHD, non-pharmacological methods (directed at the child and their environment) and pharmacotherapy are used. However, at present, pharmacotherapy for this disorder is not effective enough, and new, more effective methods of therapy are still being sought. In the presented manuscript, the authors used bioinformatic and behavioral studies for evaluating the effect of naringin in the ADHD model and the mechanism of the presented results.

In the molecular docking study, a careful literature review showed that naringin could bind to several targets, as suggested by molecular modeling and demonstrated experimentally. These biological targets include HSP90, P53, IL-6, STAT3, ESR1, BCL-2, GSK-3β, CASP3, and MMP2. Among these proteins, HSP90 and GSK-3β could be implicated in the activation of the Wnt/β-catenin signaling pathway, as previously reported [18,48]. To understand the molecular basis of the interaction of naringin with these targets, a molecular docking simulation of naringin was conducted with HSP90 and GSK-3β. The simulation showed a satisfactory binding with GSK-3β, including the critical interaction with the kinase hinge region. Many glycosides could have the ability to interact with the hinge region residues of GSK-3β, using their sugar part in a similar manner to naringin. Nevertheless, what makes naringin unique is its naringenin aglycone part, which has interesting complementarity with the hydrophobic back pocket of GSK-3β and can form an extensive network of H-bonds with its polar residues. Next, we turned our attention to investigating the potential interaction of naringin with HSP90, which is an ATP-dependent molecular chaperone that plays a key role in folding various client proteins [49]. It was obvious from the proposed docking pose that the disaccharide fragment has the perfect size for filling the polar pocket, making H-bonds with the critical Asp93 and Thr184, which similarly interact with ATP and the inhibitor JMC31. In addition, the chromene ring largely contributed to the binding via its π-stacking and hydrophobic interactions with Phe138 and Leu107, respectively. The above observations suggest the potential implication of these biological targets in the activation of Wnt/β-catenin signaling by naringin and may provide guidelines for the discovery of other bioactive glycosides with a similar mode of action.

Notably, previous studies proposed the binding of naringin into the ATP pocket of these enzymes, with the sugar moieties anchored to the critical polar residues responsible for stabilizing the ATP polar part [50,51]. The MD simulation provided more confidence in the proposed binding modes of naringin to both targets. We believe that the stable H-bonding interactions with Lys85 and Lys183 are responsible for the selectivity of naringin for GSK-3β over other kinases. Similarly, the simulation study showed that the anchoring of the sugar moiety to the critical Asp93 and Thr184 helps to stabilize the ligand in the ATP site of HSP90. These findings could encourage the search for similar flavonoids or the design of naringin derivatives to target GSK-3β and HSP90 for a variety of therapeutic applications.

These results were supported by a previously published study on the docking of naringin on GSK-3β (PDB ID: 1Q4L) using AutoDock tools, which also demonstrated the H-bond between disaccharide and Val135, in addition to the H-bond between the aglycon part and Lys 85 [52].

In our study, the bioinformatic analysis of the naringin mechanism of action was represented in the canonical Wnt signaling pathway using the KEGG database. Wnt proteins are produced and bind to their ligands, which causes stabilization of the cytoplasmic β-catenin. The β-catenin is subsequently allowed to move to the nucleus and stimulate the Wnt-regulated genes via interacting with the transcription factors and coactivators. Upon analyzing the interaction between the studied proteins using the STRING database, a network was produced with nodes representing proteins and colored edges representing different types of interactions between the proteins. The interactions include text-mining, gene co-occurrence, gene homology, interactions from curated databases, and experimentally determined interactions. The bioinformatic enrichment analysis of naringin target genes has shown a strong relation between a relatively large number of target genes and cognitive disorders like Parkinson’s disease and Alzheimer’s disease, which could indicate a relationship with other cognitive disorders, such as ADHD. Many studies support us by demonstrating that the canonical Wnt/β-catenin signaling pathway is active in the cortex during embryogenesis [53,54,55], and it controls the time and duration of neurogenesis [54,56].

The analysis and visualization of naringin and ADHD target genes and pathways showed that 13 pathways are common between the drug and the disease, which may support the relationship between naringin and ADHD. The findings of Ishii et al. suggest that naringin may facilitate the recovery of dopaminergic neurons after injury by restoring growth differentiation and neurotrophic factor (GDNF) levels in the substantia nigra and reducing Iba-1 and TNF-α levels in the striatum [57].

In the current study, behavioral parameters validated the use of SGL as a tool to induce ADHD-like symptoms in mice. The SGL group showed a significant prolongation of the grooming time in the self-grooming test, and a low percentage of mice succeeded in crawling with the rope. Furthermore, the ASST showed an increased number of required trials for the CD, RV1, and IDS criteria, implying that poor attention was noticeable in the mice fed on SGL. Similar to our results, Heisler et al. reported that animals with deficits in cognitive flexibility denoted by defective performance in the ASST need more trials to perform the tasks in the ASST [58].

Previous studies documented that oral naringin has effect on locomotion, attention, or learning capabilities per se. For example, Swamy et al. tested oral naringin 100 mg/kg in the open field test, novel object recognition test, and forced swimming test, and claimed it was inert and did not affect motor activity or cognitive function [59]. Another study, performed by Dai et al., tested the effect of oral naringin in rats in the Morris water maze, novel object recognition, and fear condition test; the authors concluded that no significant effects were observed in the animals treated with naringin 100 mg/kg [60].

The current results indicate high brain glutamate levels in the brains of mice fed with SGL. Increments in glutamatergic transmission in brain regions such as the frontal, striatal brain, and anterior cingulate cortex are linked to ADHD [61,62]. Glutamate upregulation is observed in ADHD models and other neurologic disorders [63,64]. In agreement with our study, previous rodent models of ADHD demonstrated increased brain glutamate levels in rats [33,65,66] and mice [32].

In contrast to glutamate, dopamine was found to decline in the brains of the mice fed on SGL. Similar results were obtained in animal models of ADHD based on SGL diets [32,33,66]. Dopamine deficiency is known to play a critical role in the pathology of ADHD. Imaging studies have provided additional supportive evidence of the possible involvement of catecholamine and neurotransmitter dysregulations in the etiology of ADHD. Functional imaging studies, such as positron emission tomography, which utilize selective ligands for the dopamine transporter (DAT), documented amplified DAT binding capacity in up to 70% of ADHD patients, designating a greater density of DAT in the ADHD brains than in controls [5,6]. In agreement with this, altered D2/D3 receptor availability that is responsive to methylphenidate was reported in ADHD patients [67,68]. Medications used for ADHD treatment, e.g., methylphenidate, amphetamines, and bupropion, act by inhibiting the reuptake of dopamine [69,70,71]. Furthermore, ADHD is linked to a decline in the availability of noradrenaline transporters in frontoparietal–thalamic–cerebellar regions [7]. In ADHD patients, the lack of appropriate performance in attention tasks correlates with low concentrations of urinary excreted noradrenaline metabolites [72].

In the current study, there was a reduced expression of brain Wnt/β-catenin genes and Bcl2, but overexpression of caspase 3 and BAX, in the mice fed with SGL. One study examining adult ADHD found that the KCNIP4 gene was associated with ADHD. This gene is part of a negative feedback loop within the Wnt/β-catenin pathway [73]. A connection between Wnt signaling and mental disorders was documented [74]. The Wnt signaling pathways are activated by the joining of Wnt-protein ligands to the cell-surface receptors, which are referred to as Frizzled, followed by the recruitment of Dishevelled (DVL) proteins. Indeed, the Wnt/β-catenin signaling pathway controls cell death and survival [75]. Some studies revealed a correlation between Wnt/β-catenin activation and the triggering of apoptosis, involving the overexpression of BAX and caspases-3, and the downregulation of Bcl2 [76].

The Wnt pathway contributes to Alzheimer’s disease. In brief, amyloid-β (Aβ) neurotoxicity in Alzheimer’s disease leads to downregulated Wnt signaling [77], which suggests that downregulated Wnt may be crucial in Alzheimer’s pathogenesis. Similarly, Ye et al. highlighted suppressed downstream canonical Wnt signals in senescent human cells [78] and reported that downregulated Wnt signaling stimulates senescence-linked heterochromatin focus.

Importantly, Wnt/LRP6 signaling is crucial for the regulation of axon remodeling, synaptic plasticity, and β-catenin-independent neurotransmitter release [79]. Grünblatt et al. (2018) reported activation of the Wnt/β-catenin pathway and the improvement of neuronal differentiation through treatment with methylphenidate [23]. This work shed light on the additional targets of methylphenidate and ADHD candidates. Custodio et al. provided evidence that Wnt signaling is involved in the behavioral impairment in the thyroid-hormone-responsive protein-overexpressing ADHD mouse model [22]. Caracci et al. concluded that activation of Wnt/β-catenin signaling is influential in developing new therapeutic options that may help support ADHD patients [80]. In agreement with Carcacci et al., another research group concluded that the Wnt/β-catenin pathway is disrupted in patient-specific neural stem cells [25].

The anti-inflammatory and anti-apoptotic effects of naringenin led to the assumption that it is promising for treating neurodegenerative disorders [81]. Naringenin was found to improve spatial learning and memory deficiency in the AD model; this effect was thought to be mediated through regulation of the PI3K/AKT/GSK-3β pathway and downregulation of Tau phosphorylation [82]. Further, naringenin was proven to affect apoptosis and prevent neurotoxicity. The mechanisms by which naringenin performs anti-apoptotic activity and exerts neuroprotective effects involve caspase-3-pathway inhibition, PI3K/AKT activation, and GSK-3β signaling pathway modulation [83]. Moreover, naringenin inhibits lipid peroxidation by decreasing the content of malondialdehyde in the hippocampal brain [84].

The exact mechanism of action of naringin is still to be investigated. The activities of flavonoids rely on their antioxidant and metal-chelating actions [85,86]. As the chemical structure belongs to polyphenols, flavonoids are exceptional free radical scavengers as a result of the high reactivity of their hydroxyl substituents. This free-radical-scavenging activity occurs in addition to the chelating properties of flavonoids, mediated the repressive effect of flavonoids on lipid oxidation. One study highlighted the ability of naringin to scavenge free radicals and lipid peroxides [87]. Further, naringin was described as a protectant against DNA cleavage [88]. In animal models, mice were treated with naringin prior to exposure to multiple grades of γ-radiation [36]. In addition, naringin was documented to mitigate ifosfamide-induced micronuclei in mouse bone marrow [89]. The impact of naringin on Wnt signaling has been reported in some other disorders. One of the studies on this topic investigated the probable role of Wnt/GSK-3β/β-catenin signaling in hyperthyroidism-induced disorders and explored the beneficial actions of orally administrated naringin for 2 weeks in rats with hyperthyroidism via influencing the expression of Wnt/β-catenin proteins [90].

Bcl2 is an antiapoptotic factor that acts by preventing BAX, which promotes the apoptosis of nucleus pulposus cells. Naringin at 20 ug/mL noticeably enhanced the production of Bcl2 and mitigated BAX expressions. In turn, the results revealed in this study explained how naringin was effective in suppressing apoptosis via the mitochondrial pathway [91]. This finding explains the results of the current study; an increase in gene production of Bcl2 was noticed upon treating mice with naringin 50 mg/kg and 100 mg/kg. Furthermore, decreases in Wnt, β-catenin, caspase 3, and BAX mRNA expressions were observed.

## 4. Materials and Methods

### 4.1. Investigation by Molecular Docking and Molecular Dynamic Simulation

#### 4.1.1. Molecular Docking

To understand the binding mode of naringin to its targets, we docked the compound into the X-ray crystal structures of GSK-3β (PDB ID: 4AFJ) and HSP90 (PDB ID: 8AGI), obtained from the protein databank (www.rcsb.org, accessed on 1 September 2024).

Molecular docking was performed using the FRED module implemented in OpenEye software 2023.2.3 and the Fast Rigid Exhaustive Docking method. The first step was the generation of a conformer library of naringin using the Omega module and the default settings. Next, a design unit, which comprised the prepared receptor grids, was generated using the make-receptor graphical interface provided in the OpenEye suite. The docking box was centered on the atoms of the crystallized ligands in both cases. Finally, the software performed rigid docking using the multi-conformer ligand library [92]. The 1.98 Å crystal structure of GSK-3β in complex with an oxazole inhibitor (PDB ID: 4AFJ) and the 2.10 Å crystal structure of HSP90 in complex with JMC31 (PDB ID: 8AGI) were used for docking simulation. The default scoring function of the FRED module is the Chemgauss4 function, which depends on shape complementarity. Eight types of interactions are included in the score, namely, steric, acceptor, donors, coordinating groups, metals, lone pairs, polar hydrogens, and chelator coordinating groups. Docking poses with the best scores were visually examined and the graphics were rendered using Pymol 2.5.5.

#### 4.1.2. Molecular Dynamic Simulation

Subsequently, docked poses with the best scores were further examined in complex with each enzyme through a 100-nanosecond MD simulation to gain insights into their binding determinants. Using the Desmond module in Schrodinger software (Version 2020.1), the naringin in complex with GSK-3β and HSP90 was separately solvated by a truncated octahedral box of TIP3P waters with a 12 Å distance between the farthest dimensions of the complex in each direction. The numbers of atoms were 47,508 and 29,615 in the GSK-3β and HSP90 systems, respectively. Each system was then minimized for 300 ps using the default parameters and simulated in an NPT ensemble and Nosé–Hoover thermostat for 100 ns and with a 2 fs timestep, with frames recorded every 10 ps. The trajectories were analyzed using the simulation interaction diagram within the Schrodinger software and visualized using Pymol software.

### 4.2. The Utilization of Bioinformatic Tools to Correlate the Target Protiens

A search on the KEGG database [93] was performed for exploring the mechanism of naringin and how it may affect the targeted pathway (on 3 July 2024). The input was as follows: Wnt, β-catenin, caspase 3, Bcl2, and BAX. Proteins with possible interactions with Wnt/β-catenin were studied and enriched by searching gene ontology, and pathway analysis was performed with the demonstration of the co-expressed proteins and their scores through using the String database, last accessed 20 September 2024 using the following link: (https://string-db.org/, accessed on 1 September 2024).

On 5 July 2024, the binding database was used to explore naringin targets with 0.7 similarity. Next, the naringin target genes were entered into the KEGG mapper to check the related pathways. On 8 July 2024, ADHD-related genes were retrieved from the DisGenet database using a GDA score of more than 0.4. Furthermore, naringin target genes were introduced in the ShinyGo 0.80 bioinformatic tool to make an enrichment analysis with an FDR cutoff of 0.05 on 11 July 2024 to look for a relation between the drug and the disease.

On 18 July 2024, we utilized the FunRich 3.1.3 bioinformatic tool for visualization and analysis of target genes and pathways. In addition, a Venn diagram was created for the shared pathways between naringin and ADHD. In addition, a network of the naringin target genes and pathways from one side and ADHD from another side was created using Cytoscape 3_10_2 on 19 July 2024.

### 4.3. The Mouse In Vivo Study

#### 4.3.1. Chemical Preparation

Monosodium glutamate (SGL) was procured from Algomhoria Company (Cairo, Egypt) and included in a fixed portion mixed with a standard diet (0.4 g/kg). Naringin (C27H32O14) was obtained as a white powder from Alfa Aesar (ThermoFisher Company, GmbH, Dreieich, Germany), prepared as a suspension in 1% CMC aqueous solution in distilled water, and given to mice by an oral gavage tube.

#### 4.3.2. Mouse Environment and Housing Conditions

Male Swiss albino mice (3 to 4 weeks old, with a body weight range of 9–15 g) were kept in polyethylene cages in groups of six mice. Mice were maintained in the animal facility at Suez Canal University. A normal D/L cycle was maintained (13 h for the lighting phase and 11 h for the dark phase at that time of the year). Free access to the prepared food mixture and clean tap water were provided to the mice throughout the experiment. The experimental procedures received approval from the Medicine Ethical Committee at Suez Canal University (5690#).

#### 4.3.3. Experimental Design

The male Swiss albino mice were divided equally and randomly into four experimental groups with different dietary compositions and drug treatments, as shown in Table 1. The normal chow diet given to Group 1 was composed mainly of crushed yellow corn, whereas the SGL diet was composed of the same normal chow diet—like that administered to Group 1—but mixed with 0.4 g of SGL for every 1 kg of normal diet.

#### 4.3.4. Evaluation of Mouse Psychomotor Activity by Behavior Tasks

##### Self-Grooming Test

The mice were observed for impulsive grooming activities, such as face rubbing and licking or biting paws and fur, following a previous report [96], with some modifications. Each mouse was placed into a cage with dimensions equal to 38 cm × 25 cm × 23 cm for an assessment period of 10 min [97].

##### Rope Crawling Test

During this test, each mouse was suspended for three minutes on a 2 m long rope positioned 150 cm above the floor of the room (altitude stress) and observed for their responses. The quantification of mice that were able to grab or crawl down the hung rope in a “successful” manner was performed, but the mice that were unable to crawl up the rope or grasp it securely were considered to have “failed” this test. Both criteria were quantified. The success rate of each experimental group was calculated for statistical analysis [98].

##### Attentional Set-Shifting Task (ASST)

**Figure 12:**
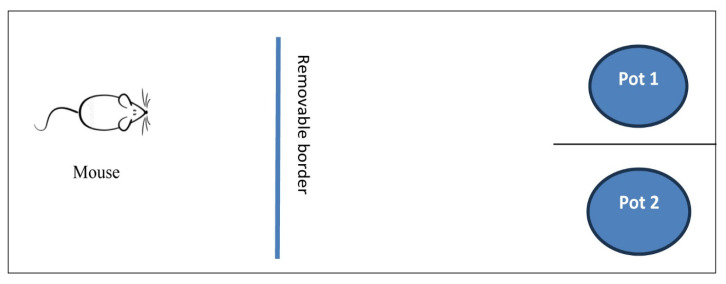
A diagram of the attention set-shifting task (ASST) for mice.

The ASST was performed following a previously published protocol [99]. The testing arena was rectangular and made of plexiglass with the following dimensions, 40 × 80 × 30 cm. The task contained 2 transferable plates dividing the arena into equal thirds (by length). The first third, on the left side, was considered the starting box, and there was a removable panel placed in the right third to divide it into two equal sections; each section had a pot (Figure 12).

- The Habituation Phase (5 Days)

Each mouse was trained for 20 min/day to freely explore the arena without any testing stimuli for 5 days (with a restricted fixed basal diet not leading to loss of more than 15% of their body weight). A food reward (which was a piece of cereal) was introduced to the cage to make it familiar to the mouse (regarding color and odor).

- The Training Phase (2 Days)

Training was performed on days 6 and 7 (2 training days). To obtain a food reward in the pot (without filling), 3 successful attempts were required. The mouse was trained on digging in the pots media to obtain the food reward embedded in cage bedding.

Mice were empowered to detect the presence/absence of the food reward (cereal) in the pot by either an olfactory stimulus (odor related to the digging medium) or a tactile stimulus (texture of the digging medium). The training was achieved by enabling the mice to dig for the food reward until finishing 6 consecutive correct trials with the accurate choice for the pot. Animals that failed to dig for food rewards within 2 h from the start of training were excluded. The examples used in training were not used again for testing.

- Experimentation Phase (1 Day)

On day 8 (the experimentation day), the first step in training was simple discrimination (SD) between the pots, which were different in one aspect (either an olfactory stimulus or a tactile stimulus). After completing the required training, the mice were shifted to another test for compound discrimination (CD), in which the familiar pots were used in the SD with a similar reward aspect, but after introducing a second, non-relevant aspect.

After the mice learned the assigned training idea, the reinforcement rules were revised, and the mice were trained to identify that the originally exact stimulus within the rewarded aspect had become “not correct” during this phase; this stage was designated as reversal-1 (RV1). After mice accomplished the criterion for RV1, they were moved to a new testing idea. Intra-dimensional shift (IDS) was achieved by presenting the mice with a new group of stimuli, but they were commanded to pay attention to the same perceptual aspect reinforced during the SD, CD, and RV1. The test was completed at the start of the dark phase of the day (5–7 pm).

In summary, a 3 min trial was allowed to determine each mouse choice. In the waiting area, mice had free access to water to offer a chance to drink and prevent thirst. In the experimentation phase, the total number of trials made by each mouse for reaching the criterion set with each stage was registered and compared.

#### 4.3.5. Mouse Scarification

The mice received an injection of ketamine (85 mg/kg) [100] and were then sacrificed by cervical dislocation. Next, the brains were dissected, blood was washed out, and the brains divided into 2 separate hemispheres. The right hemisphere was fixed in 4% paraformaldehyde solution to be used later for histological staining [100], and the left brain hemisphere was subjected to immediate freezing at −80 °C.

#### 4.3.6. Molecular Analysis

Homogenized brain tissues (15 mg) were used to extract total RNA. This was performed using the miRNeasy mini kit purchased from QIAGEN (CAT. NO.217004, Hilden, Germany). Next, complementary DNA was obtained after converting RNA to cDNA utilizing QuantiTect Reverse Transcription Kit obtained from QIAGEN company (CAT. NO.205311, Germany). Finally, gene analysis for Wnt, β-catenin, caspase-3, Bcl2, and BAX was computed in StepOne real-time PCR instrument from Thermo Scientific Company (CAT. NO. 4376357, Oxford, UK). The reaction volume included 10 μL of HERA SYBR^®^ Green qPCR master mix (Willowfort, Birmingham, UK) and 15 pmol from primer pairs, as shown in Table 2, and 150 ng of cDNA, and the conditions used were initial denaturants at 95 °C for 5 min session, followed by 40 cycles of denaturant 95 °C for half a minute, annealing at 58–60 °C for the 30 s, and extension at 72 °C for 30 s. The mRNA fold changes for the target genes were determined by applying the 2^−ΔΔCt^ method [101], and normalized gene expression was achieved relative to the β-actin gene.

#### 4.3.7. Assessment of Inflammatory Mediators

Brain tissues were homogenized in RIPA buffer (Sigma-Aldrich Chemie Gmbh, Buchs, Switzerland) and homogenates were cleared via centrifuging at 1500× *g*. ELISA kits for dopamine (Catalog # MBS732020), glutamate (Catalog # MBS756400nd), TNF-α (Catalog # MBS825075), and NFκB (Catalog # MBS043224) were used in this study. The kits were purchased from MBS Company (San Diego, CA, USA) and the measurement of the reaction products was performed at 450 nm.

#### 4.3.8. Histopathology, Immunohistochemical Staining, and Examination

The right hemispheres were formalin-fixed, and then implanted in liquid paraffin, and were allowed to cool. Sections (4 µm in thickness) were prepared from the paraffin blocks. For routine examination, sections were cut and stained with hematoxylin and eosin to show the neuron cell body arrangement and the appearance of the nuclei and the fibrillary cytoplasmic processes [64,102].

Other sections were allowed to dry and then subjected to immunohistochemical staining for Bcl2 using rabbit polyclonal antibodies (cat # A16776, ABclonal, Swansea, UK). Next, the horseradish peroxidase label was applied for 1 h, followed by DAB chromogen for 14 min using the Mouse/Rabbit PolyDetector detection system (Cat# BSB0205, Bio SB, Goleta, CA, USA). Mayer’s hematoxylin was utilized to counterstain the tissues. All slides were imaged at 400×, using the Leica Microsystems. A calibrated standard digital microscope camera was fixed to a Leica microscope (Leica model DM 1000, Heidelberg, Germany), with 10 megapixel resolution (3656 × 2740 pixels). Morphometric comparison for the color area in each section was set [103] for Bcl2 in the hippocampus by alienating the area stained with DAB from hematoxylin and converting the color information to red, green, and blue (RGB) images with multiple stains [104].

#### 4.3.9. Statistical Analysis and Data Manipulation

Data were collected by the authors, tabulated, and then presented as the mean and standard deviation. Comparison between groups was achieved by application of the one-way ANOVA test and Bonferroni’s test at *p* < 0.05 considering all possible comparisons among the study groups. Quantal data were analyzed using the Chi-squared test.

## 5. Conclusions

In conclusion, the bioinformatic study indicated that Wnt/β-catenin is involved in the naringin mechanism of action; this is a common pathway in neurologic disorders. Furthermore, molecular docking indicated the possible inhibition of GSK-3β and HSP90 by naringin.

The mouse study highlighted that feeding mice with SGL for eight weeks produced behavioral signs of ADHD in these mice; poor attention was observed in the ASST and rope crawling test, and locomotor hyperactivity was observed in the grooming test. The brains showed downregulated levels of Wnt/β-catenin and Bcl2 proteins and upregulated inflammatory mediators. In mice cotreated with naringin, the behavioral signs of ADHD were diminished, and the inflammatory markers were downregulated. In addition, upregulation of Wnt/β-catenin and Bcl2 proteins and downregulation of caspase 3 and BAX were observed. These results open avenues for more studies on the role of naringin in ADHD models to fully elucidate its mechanism of action.

## Figures and Tables

**Table 1:** The experimental groups in the mouse study.

Number	Name	Applied Diet	Treatment
Group 1	Normal control	Normal chow diet *	Distilled water
Group 2	SGL control	SGL diet ^†^ [94]	Distilled water
Group 3	SGL + naringin-50 mg/kg	SGL diet	Naringin 50 mg/kg [95]
Group 4	SGL + naringin-100 mg/kg	SGL diet	Naringin 100 mg/kg [59,60]

* The normal chow diet was composed mainly of crushed yellow corn. ^†^ The SGL diet was composed of SGL (0.4 g/kg) for each 1 kg of the normal chow diet [94].

**Table 2:** Primer sequences for target genes.

GenesPrimers	Sequences	Accession Number
*Wnt*	Forward 5′GCCTGTGAAGGACTCAGAACTTG3′ Reverse 5′AGCTGTCACTGCCGTTGGAAGT3′	NM_001285794.1
*β-Catenin*	Forward 5′GTTCGCCTTCATTATGGACTGCC3′ Reverse 5′ATAGCACCCTGTTCCCGCAAAG3′	NM_007614.3
*Caspase-3*	Forward 5′GGAGTCTGACTGGAAAGCCGAA3′ Reverse 5′CTTCTGGCAAGCCATCTCCTCA3′	NM_009810.3
*Bcl2*	Forward 5′CCTGTGGATGACTGAGTACCTG 3′ Reverse 5′AGCCAGGAGAAATCAAACAGAGG3′	NM_009741.3
*BAX*	Forward 5′AGGATGCGTCCACCAAGAAGCT3′ Reverse 5′TCCGTGTCCACGTCAGCAATCA3′	NM_007527.3
*β-actin*	Forward 5′TCCTCCTGAGCGCAAGTACTCT3′ Reverse 5′GCTCAGTAACAGTCCGCCTAGAA3′	NM_007393.5

## Data Availability

The original contributions presented in this study are included in the article. Further inquiries can be directed to the corresponding author.
